# Detection of Small *CYP11B1* Deletions and One Founder Chimeric *CYP11B2/CYP11B1* Gene in 11β-Hydroxylase Deficiency

**DOI:** 10.3389/fendo.2022.882863

**Published:** 2022-05-24

**Authors:** Hua Xie, Hui Yin, Xue Ye, Ying Liu, Na Liu, Yu Zhang, Xiaoli Chen, Xiaobo Chen

**Affiliations:** ^1^ Department of Medical Genetics, Capital Institute of Pediatrics, Beijing, China; ^2^ Department of Endocrinology, Affiliated Children’s Hospital of Capital Institute of Pediatrics, Beijing, China; ^3^ Bioinformation Department, Beijing Mygenostics Co., Ltd, Beijing, China; ^4^ Department of Laboratory Center, Capital Institute of Pediatrics, Beijing, China

**Keywords:** 11β-hydroxylase deficiency, molecular diagnosis, chimeric CYP11B2/CYP11B1 gene, founder rearrangement, small deletion

## Abstract

**Objective:**

11β-Hydroxylase deficiency (11β-OHD) caused by mutations in the *CYP11B1* gene is the second most common form of congenital adrenal hyperplasia. Both point mutations and genomic rearrangements of *CYP11B1* are important causes of 11β-OHD. However, the high degree of sequence identity between *CYP11B1* and its homologous gene *CYP11B2*, presents unique challenges for molecular diagnosis of suspected 11β-OHD. The aim of this study was to detect the point mutation, indel, small deletion of *CYP11B1* and chimeric *CYP11B2*/*CYP11B1* gene in a one-tube test, improving the genetic diagnosis of 11β-OHD.

**Methods:**

Optimized custom-designed target sequencing strategy was performed in three patients with suspected 11β-OHD, in which both the coverage depth of paired-end reads and the breakpoint information of split reads from sequencing data were analysed in order to detect genomic rearrangements covering *CYP11B1*. Long-range PCR was peformed to validate the speculated *CYP11B1* rearrangements with the breakpoint-specifc primers.

**Results:**

Using the optimized target sequencing approach, we detected two intragenic/intergenic deletions of *CYP11B1* and one chimeric *CYP11B2*/*CYP11B1* gene from three suspected patients with 11β-OHD besides three pathogenic heterozygous point mutation/indels. Furthermore, we mapped the precise breakpoint of this chimeric *CYP11B2*/*CYP11B1* gene located on chr8:143994517 (hg19) and confirmed it as a founder rearrangement event in the Chinese population.

**Conclusions:**

Our optimized target sequencing approach improved the genetic diagnosis of 11β-OHD.

## Introduction

Congenital adrenal hyperplasia (CAH) is one of the most common inherited endocrine diseases and is caused by a deficiency in enzymes required for the synthesis of cortisol from cholesterol (1). In general, all forms of CAH are transmitted in an autosomal recessive mode of inheritance as a monogenic disorder. 21-Hydroxylase deficiency is the most common cause of CAH, accounting for 90–99% of cases (2). In contrast, 11β-hydroxylase deficiency (11β-OHD) is the second leading cause of CAH, accounting for approximately 5-8% of cases (3). CAH due to 11β-OHD has an overall prevalence of 1 in 100,000 live births (4).

11β-OHD leads to high levels of 11-deoxycortisol and 11-deoxycorticosterone, which are shunted into adrenal androgen synthesis pathways, with high levels of androgenic steroids and androstenedione. Profound virilization and significant masculinization of the external genitalia is thus exhibited in female newborns (5). Sexual precocity, rapid somatic growth, and rapid skeletal maturation due to hyperandrogenaemia are exhibited in both untreated males and females with 11β-OHD, leading to short stature in adulthood (6, 7). Hypertension occurs in approximately two-thirds of cases and often occurs early in life due to the accumulation of the potent mineralocorticoid 11-deoxycorticosterone (8).

11β-Hydroxylase is encoded by *CYP11B1* (OMIM # 610613), 40 kb apart from its homologic gene *CYP11B2* (OMIM #124080), on chromosome 8q21-22 (9). *CYP11B1* and *CYP11B2* both consist of nine exons and share 95% sequence homology in the exons and 90% sequence homology in the introns; however, they have distinct functions (10). More than 100 mutations in *CYP11B1* have been reported to date, most of which are point mutations (11–14). Moreover, the chimeric *CYP11B2/CYP11B1* gene has been confirmed to be an important cause of 11β-OHD, mostly occurring in introns 2 and 6 of *CYP11B2* (15, 16). In this autosomal recessive disorder, when only a heterozygous pathogenic point mutation of *CYP11B1* is identified in patients with signs of 11β-OHD by sequence analysis, the focus of the search for the second allele, such as intronic nonclassical splicing variants, intragenic/intergenic deletions or a chimeric *CYP11B2/CYP11B1* gene, is necessary.

With the extensive application of next-generation sequencing (NGS) in the clinic, copy number variation (CNV) detection algorithms from NGS data have been developed quickly (17–23). Some algorithms are based on the coverage depth of capture sequencing data to detect CNVs (19, 21, 22). These coverage depth-based algorithms can detect most CNVs >200 kb. However, their ability to detect smaller CNVs becomes much less reliable, especially for small intragenic CNVs (<10 kb), single-exon CNVs or CNVs containing homologous gene families or pseudogene families. To address this unmet need, several optimized CNV detection algorithms combining the coverage depth of paired-end reads with misalignment information of split reads indicative of genomic rearrangement have been developed (24). The application of NGS in detecting small *CYP11B1* deletion or chimeric *CYP11B2/CYP11B1* rearrangement has not been reported.

In the present study, we used our custom-designed target sequencing strategy to detect the point mutation and indel of *CYP11B1* in parallel with genomic rearrangement for patients suspected to have CAH due to 11β-OHD, and both the coverage depth of paired-end reads and the breakpoint information of split reads from target sequencing data were analysed to detect genomic rearrangement covering *CYP11B1*. We identified three CAH patients due to *CYP11B1* rearrangement, including one intragenic deletion, one intergenic deletion, and one previously reported chimeric *CYP11B2/CYP11B1* gene. Furthermore, we decoded the precise breakpoint sequence of these *CYP11B1* rearrangements and confirmed this chimeric *CYP11B2/CYP11B1* gene with a breakpoint located on chr8:143994517 (hg19) in intron 6 of *CYP11B2* as a founder rearrangement event in the Chinese population. Our optimized target sequencing approach improves the genetic diagnosis of CAH due to 11β-OHD, and our data are useful for genetic counselling.

## Materials and Methods

### Clinical Studies

All clinical investigations and genetic analyses were performed according to the guidelines of the Declaration of Helsinki. This study was approved by the ethics committee of the Capital Institute of Pediatrics (SHERLL 2018005). Written informed consent was obtained from all the patients’ guardians/parents/next of kin for the publication of this clinical information.

### Target Gene Enrichment, Library Preparation of the DNA Template, and Sequencing

Genomic DNA was isolated from peripheral leukocytes using a DNA isolation kit (Tiangen, Beijing, China) according to the manufacturer’s instructions and then captured by a commercial NGS kit (MyGenostics GenCap Enrichment Technologies, Beijing, China). Whole coding and flanking intronic regions (50 bp) of 276 genes associated with adrenal disease were captured (**Supplementary Table 1**). The enriched libraries were sequenced using an Illumina HiSeq X Ten sequencer (Illumina, San Diego, USA) for paired reads of 150 bp. Cluster generation and sequencing were carried out according to the manufacturer’s instructions.

### Analysis of Target Sequencing Data

Illumina sequencing adapters and low-quality reads (<80 bp) were filtered out, and the clean reads were mapped to the UCSC hg19 human reference genome. The mapped sequences were then processed using GATK software (https://software.broadinstitute.org/gatk), and SNPs and indels were detected by GATK HaplotypeCaller. After excluding common variants (1% of the public databases, such as dbSNP, 1000 Genomes Project and gnomAD), the candidate variants were retained using the QIAGEN^®^ Clinical Insight (QCI^®^) Interpret Translational tool (https://apps.qiagenbioinformatics.cn/). The functional effects of missense mutations were predicted by four algorithms (PolyPhen, SIFT, REVEL and Mutation Taster). The inheritances of candidate variants were validated in core family members *via* Sanger sequencing. The pathogenicity of each variant was interpreted according to the guidelines of the American College of Medical Genetics and Genomics (ACMG) (25).

CNVs were identified using CNVkit (26) based on the read-depth algorithm and DELLY (27) based on the split-read and paired-end algorithms. Normal references used for CNV identification were constructed using the sequencing data in the same run and same capture panel. Default settings were used for CNV identification individually. CNVs detected from the generated BCF file were further filtered using the “Delly filter” function to exclude poor ones. Sequence coverage and examination of the split reads were visualized in Interactive Genome Viewer (IGV) developed by the Broad Institute (28), using the setting of “show soft-clipped bases”.

### Long-Range PCR and Breakpoint Mapping

Based on the genomic location from paired-end reads and the misalignment sequence information from split reads, we designed breakpoint-specific primers (**Supplementary Table 2**) for long-range PCR (Platinum PCR SuperMix High Fidelity kit, Invitrogen, CA) to validate the speculated *CYP11B1* rearrangement. Sanger sequencing (ABI 3700) was performed to decipher the characteristics of the breakpoint. In addition, we attempted *CYP11B1-*specific long-range PCR to validate the point mutation and indel of *CYP11B1* with primers that have been previously reported (29) (**Supplementary Table 3**).

## Results

### Case Reports

The clinical features and laboratory evaluations of the three patients are listed in **Tables 1**, **2**, respectively. All patients were from nonconsanguineous families. Karyotype analysis revealed that patient 1 and patient 3 were 46, XY while patient 2 was 46,XX. 11β-OHD was diagnosed based on the clinical features and biochemical characterizations.

**Table 1:** Clinical characteristics of three patients with 11β-OHD.

Patient	Age	Sex of rearing	Karyotype	Height (cm)	Weight (kg)	Bone Age (y)	Blood pressure (mmHg)	Prader score, Penis (cm), Testis (ml)	Therapy
P1	6y	Male	46, XY	139 (+4.5SD)	29.5 (+2.9SD)	12.5	124/94 (>P99)	5, 8, 8/8	Hydrocortisone, Spironolactone
P2	2y	Female	46, XX	97 (+3.0SD)	14.9 (+2.3SD)	7	124/73	3, 2, -	Hydrocortisone
P3	11y	Male	46, XY	159 (+2.0SD)	71.9 (+4.5SD)	17	160/100 (>P99)	5, 7, 6/6	Hydrocortisone, Spironolactone, Captopril

**Table 2:** Biochemical characterization of three patients with 11β-OHD.

Patient	K+ (mmol/l)	Na+ (mmol/l)	Cortisol (ug/dl)	ACTH (pg/ml)	T (nmol/l)	P (nmol/l)	LH (IU/l)	FSH (IU/l)	17-OHP (ng/ml)	AD (nmol/l)	DHEA (ng/ml)	11-deoxycortisol (pg/ml)	Aldosterone (pg/ml)
P1	3.45	144	2.33	496	17.49	6.55	0.29 (≤1.4)	0.76 (≤3.1)	NA	NA	NA	NA	14.9
P2	3.27	145	2.59	433.9	8.57	6.93	0.25 (≤0.4)	1.32 (≤7.6)	10.77	69.29 (≤1.78)	23.16 (1.2-6.3)	198512.4	5.486
P3*	2.52	142	1.05	3	11.45	6.28	17.9 (≤7.8)	14.49 (≤4.6)	18.12	12.22 (≤7.71)	15.18 (1.7-6.1)	42176.1	42.88
Reference value	3.5-5.5	135-145	6.2-18.1	7.2-63.3	≤2.37	≤0.64	–	–	0.31-2.3	–	–	<3440.0	30-160

*after treatment; NA, not available.

#### Patient 1

**Figure 1:**
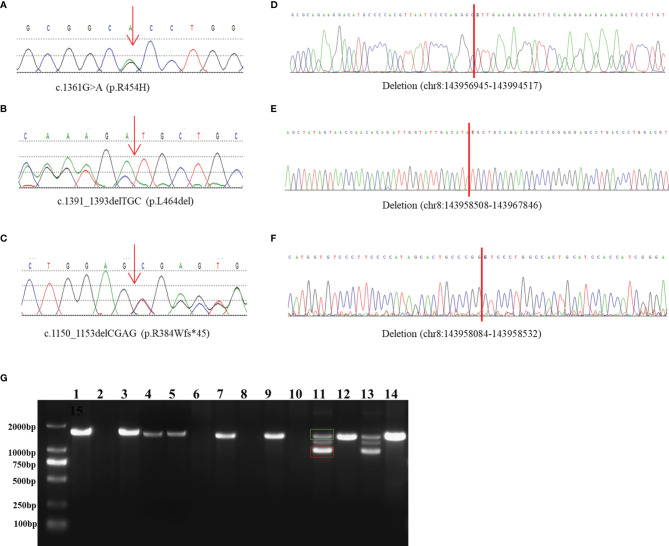
Identification and characterization of rearrangements in three patients. **(A–C)** Sanger traces for *CYP11B1*-specific PCR products show the point mutation and indels in patient 1 **(A)**, patient 2 **(B)**, and patient 3 **(C)**. **(D–F)** Sanger traces for breakpoint-specific PCR products for *CYP11B1* rearrangements. They show the sequences around breakpoints in patient 1 **(D)**, patient 2 **(E)** and patient 3 **(F)**. Red lines indicate the location of genomic rearrangement. **(G)** Electrophoretogramof PCR products for the *CYP11B1* rearrangements detected in three patients. Lanes 1–6: *CYP11B2/CYP11B1* allele in patient 1, his father, his mother, carrier 1, carrier 2, and control, respectively. Lanes 7–10: *CYP11B1* deletion fragment in patient 2, her father, her mother, and control, respectively. Lanes 11–14: The wild-type allele (green box) and the deletion allele (red box) of *CYP11B1* in patient 3, his father, his mother, and the control, respectively.

The patient was a 6-year-old boy who was admitted to our hospital for accelerated growth (23cm within one year) and penile enlargement. He presented with hypertension, excessive skin darkness, accelerated growth velocity, acne and precocious puberty. On physical examination, he presented with Tanner II pubic hair, penis of 8 cm in length, and testicular volume of 8ml for the two sides. Imaging examination showed hyperplastic adrenal glands and advanced bone age (12.5y). He was given hydrocortisone replacement (13mg/m2/d) as well as spironolactone for his uncontrolled severe hypertension (124/94mmHg, >P99). During follow-up visits, he achieved his final height (168cm, SDS -0.77) when he was 12 years old, which was 3cm lower than his midparental height, and the hypertension was well controlled. One heterozygous missense variant (NM_000497.3:c.1361G>A) was detected in *CYP11B1* (**Figure 1A**), which is a hotspot variant in Han Chinese population.

#### Patient 2

This 23-month-old girl was referred to our hospital because he was born with ambiguous genitalia, accompanied by accelerated growth and hyperpigmentation. The physical examination showed external genetalia were Prader III with 2cm phallus and no palpable gonads. Her high blood pressure was 124/73mmHg, which was much higher than the blood pressure for normal children younger than 3-year-old (100/60mmHg) (30). A computed tomography scan showed bilateral adrenal enlargement. Pelvic ultrasound revealed a normal uterus and ovaries. Following hydrocortisone treatment (12mg/m2/d), adrenal androgen concentrations were successfully normalized, as was her blood pressure. One novel heterozygous in-frame variant (NM_000497.3:c.1391_1393delTGC) of *CYP11B1* was detected (**Figure 1B**).

#### Patient 3

This 11-year-old boy was diagnosed with CAH at a local hospital due to skin hyperpigmentation and penile enlargement. However, his blood pressure and hyperandrogenaemia were not well controlled despite a high dose of hydrocortisone replacement (20mg/m2/d). Triptorelin was also administered to depress the hypothalamus-pituitary-gonadal axis. He was admitted to our hospital at the age of 11 years old with complaints of headache, stomach ache, vomiting, fatigue, hypertension (160/100mmHg, >P99) and hypokalaemia (serum potassium 2.52mmol/l). Physical examination showed that he had already achieved his final height (159cm), which was significantly shorter than his midparental height (180.5cm) (31). His pubic hair was Tanner V, and his testicular volume was Tanner II (6mL). Imaging examination showed bilateral adrenal nodular thickening and advanced bone age (17 years old). Although we gave him high doses of hydrocortisone (35mg/m2/d) combined with captopril and spironolactone, his blood pressure was maintained at the upper limit of normal range. One heterozygous frameshift variant (NM_000497.3:c.1150_1153delCGAG) was detected in *CYP11B1* (**Figure 1C**), which resulted in a truncated protein of 11 β-hydroxylase with a loss of 73 amino acids and has been reported previously in one Chinese patient (14).

### Three *CYP11B1* Rearrangements Detected by Target Sequencing

**Figure 2:**
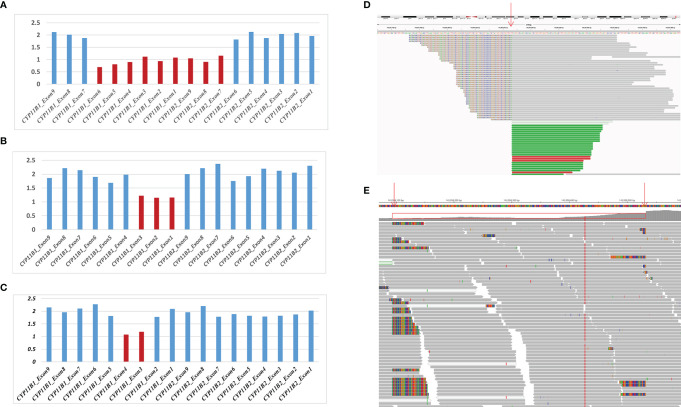
Detection of hemizygous intergenic/intragenic deletions in target sequencing data. **(A–C)** Intergenic/intragenic deletions in three patients. **(A)** Heterozygous deletion of exons 1-6 of *CYP11B1* and exons 7-9 of *CYP11B2* in patient 1 suggesting the chimeric *CYP11B2/CYP11B1* gene. **(B)** Heterozygous deletion of exons 1-3 of *CYP11B1* in patient 2. **(C)** Heterozygous deletion of exons 3-4 of *CYP11B1* in patient 3. The *x*-axis represents the corresponding exons in *CYP11B1* or *CYP11B2*. The *y*-axis shows the ratio of the mean coverage of the test sample to that of the control samples. **(D, E)** Misalignment information of split reads indicate the *CYP11B1* rearrangements shown by IGV. **(D)** The red arrow indicates the breakpoint of chr8:143967846 in patient 2. **(E)** The red arrows indicate the breakpoint of chr8:143958084 and chr8:143958532 in patient 3.

Based on the sequencing coverage depth of target regions of *CYP11B1* and *CYP11B2* (NM_000498), three speculative *CYP11B1* rearrangements with different sizes were screened out (**Figure 2**). For patient 1, in addition to the deletion covering exons 1-6 of *CYP11B1*, a deletion covering exons 7-9 in *CYP11B2* was detected (**Figure 2A**). Patient 2 carries one intergenic deletion covering exons 1-3 of *CYP11B1* (**Figure 2B**), and patient 3 carries one intragenic deletion covering exons 3-4 of *CYP11B1* (**Figure 2C**). Furthermore, the split reads of the target regions, particularly of these speculated deletions, were extracted to confirm the reality of the deletions. Clear and long misaligned sequences were seen at the arrangement breakpoints by IGV, even at the base pairs corresponding to the genomic coordinates of *CYP11B1* (**Figures 2D, E** and **Supplementary Figures 1, 2**).

### Deletion Validation and Sequence Analysis in Breakpoint-Specific PCR

The amplified PCR products were visualized on an agarose gel (**Figure 1G**) and sequenced using the breakpoint-specific primers (**Supplementary Table 2**) on 3700 ABI DNA Analyzer. Alignment of the sequencing data confirmed the genomic coordinates of the deletion (chr8:143956945-143994517, 37.6 kb, hg19) in patient 1 containing the upstream region and exons 1-6 of *CYP11B1* and exons 7-9 of *CYP11B2*, which forms a *CYP11B2/CYP11B1* chimeric that located on chr8:143994517 (hg19), in the intron 6 of *CYP11B2* (**Figure 1D**). The intergenic *CYP11B1* deletion (chr8:143958508-143967846, 9.3 kb, **Figure 1E**) in patient 2 covers both the upstream regions and exons 1-3 of *CYP11B1*, while the intragenic *CYP11B1* deletion (chr8:143958084-143958532, 448 bp, **Figure 1F**) in patient 3 covers exons 3-4 of *CYP11B1*. The genomic locations from breakpoint-specific PCR were all consistent with the speculated coordinates of the split reads from target sequencing data.

**Figure 3:**
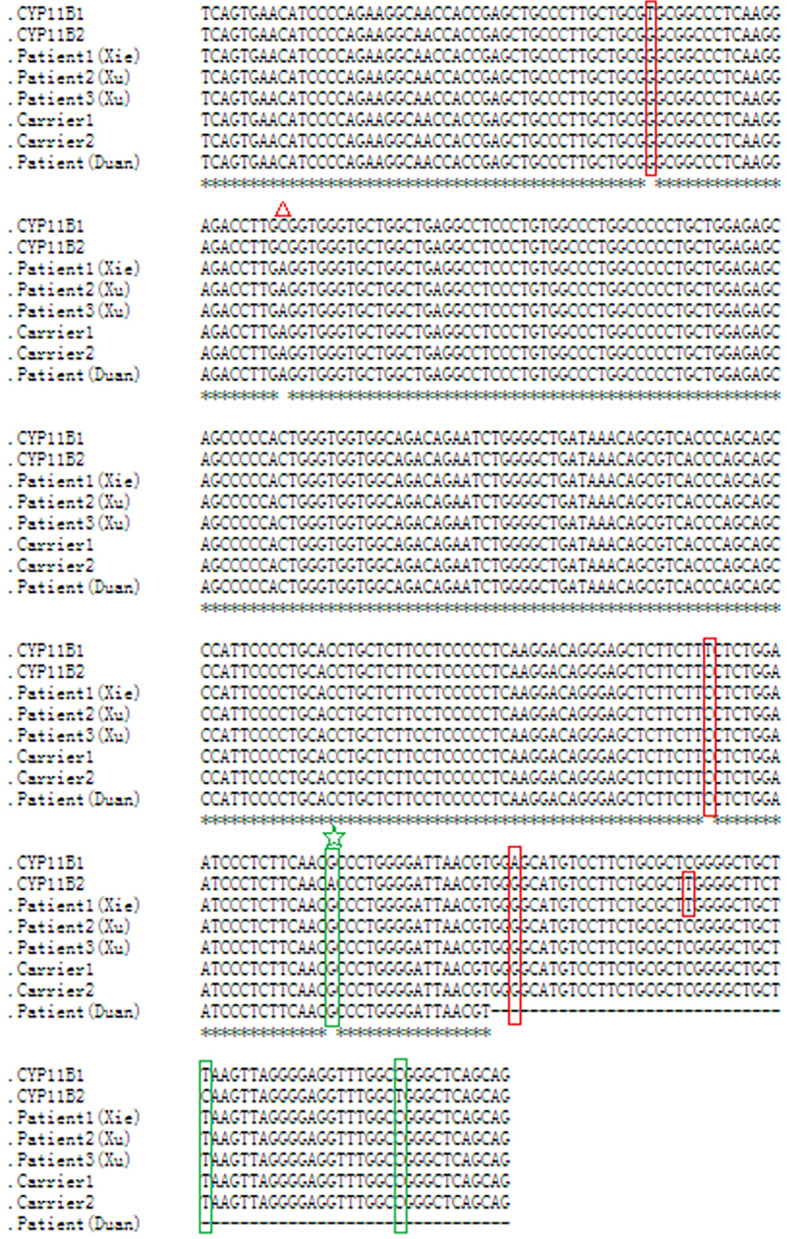
Nucleotide sequence alignment of the chimeric *CYP11B2/CYP11B1* gene. Sequence alignment of *CYP11B1* and *CYP11B2*, and the chimeric *CYP11B2/CYP11B1* gene detected from patient 1, carrier 1 and carrier 2 in our study, patient 2 and patient 3 in Xu’s study, and the patient in Duan’s study using the online Clustal W method (http://www.ebi.ac.uk/Tools/msa/clustalo/). Red boxes indicate *CYP11B2*-specific SNPs, and green boxes indicate *CYP11B1*-specific SNPs. The red triangle represents a polymorphism in *CYP11B2*. The same breakpoint in four patients and two carriers is highlighted with the green star. The black horizontal lines represent the missing downstream sequences in the patient in Duan’s study.

As this chimeric *CYP11B2/CYP11B1* gene in our study has a similar breakpoint to that of three reported Chinese patients (29, 32), we retrieved their detailed genomic sequences around the breakpoints and found that three of these patients (patient 2 and patient 3 in Xu’s study and the patient in Duan’s study) had identical breakpoint as patient 1 in our study (genomic coordinate is chr8:143994517, **Figure 3**). We also analysed the sequence characteristics surrounding the breakpoints and revealed nearby microhomologies (CTCTGGAATCCCTCTTCAAC in patient 1 and GGCCAGGGAC in patient 3) (**Table 3**). The DNA replication fork stalling and template switching (FoSTeS)/microhomology-mediated break-induced replication (MMBIR) mechanism can generate genomic, genic and exonic complex rearrangements in humans (33). Our results also suggested that the MMBIR model underlies both intergenic and intragenic deletion of *CYP11B1*.

**Table 3:** Genetic characteristics of three patients with 11β-OHD.

ID	Variation of *CYP11B1*	Inheritance	Size of Deletion	Sequence profile around breakpoints ^a^	Microhomology (Bases)
P1	Deletion (chr8:143956945-143994517)	Maternal	37.6kb	GGGAGCTCTTCTTCCTCTGGAATCCCTCTTCAACccctggggatt……gggagctcttctttctctggaatccctcttcaacGCCCTGGGGATTAA	20
	NM_000497.3:c.1361G>A	Paternal			
P2	Deletion (chr8:143958508-143967846)	Maternal	9.3kb	GATTGGTATTGA ***CA*** TAaaaacagaca……ccaggccctgaagaagaagg ***TG*** CTGCAGAACGCCCGGGGG	none
	NM_000497.3:c.1391_1393delTGC	Paternal			
P3	Deletion (chr8:143958084-143958532)	Maternal	448bp	TGCAGTGGCCAGGGACttctcccaggccctg……ggtgaggccagggacCCGGGCAGTGCTATGGGG	10
	NM_000497.3:c.1150_1153delCGAG	Paternal			

a Small letters are deleted sequences, capital letters on each side are remaining sequences, underlined nucleotides indicate microhomology, and bold italic nucleotides are complementary.

### Point Mutation and Indel Validation and Family Segregation Analysis

In the three patients, the point mutation and indels of *CYP11B1* were all inherited from their fathers, and the *CYP11B1* rearrangements were all inherited from their mothers (**Table 3**).

## Discussion

*CYP11B1* and *CYP11B2* encoded homologues, and have distinct functions in cortisol and aldosterone synthesis, respectively (34). The high degree of sequence identity between *CYP11B1* and *CYP11B2* presents unique challenges for molecular diagnosis of 11β-OHD. In previous studies, molecular genetic testing for *CYP11B1* mutations was mostly based on *CYP11B1-*specific PCR with the help of several key SNPs between *CYP11B1* and *CYP11B2* (16, 29, 32, 35). Although this method of analysis can reliably detect small genetic lesions, including the point mutation and small indel, it does not easily detect intragenic/intergenic deletion or chimeric *CYP11B2/CYP11B1* gene, which are important causes of 11β-OHD (15, 16). Menabò S used homemade MLPA probes to identify a novel chimeric *CYP11B2/CYP11B1* gene in a Sicilian patient (36); however, this MLPA method has not been widely used in genetic laboratory. MacKenzie reported a method of genotyping *CYP11B1* and *CYP11B2* for common polymorphisms and quantification of their respective mRNAs through specific real-time RT–PCR (37). Our optimized CNV detection algorithms from target sequencing can accurately detect the point mutation, indel, small deletion (even 448 bp), and even the chimeric *CYP11B2/CYP11B1* gene in a one-tube test. The ability to detect different categories of *CYP11B1* arrangement proved its application value in the clinical molecular diagnosis of CAH due to 11β-OHD.

By reviewing previous reports about the chimeric *CYP11B2/CYP11B1* gene, we collected ten patients with the chimeric *CYP11B2/CYP11B1* gene (15, 16, 29, 32, 38, 39), five of whom had the chimeric *CYP11B2/CYP11B1* gene located in intron 6 of *CYP11B2*. Interestingly, the breakpoints of the chimeric *CYP11B2/CYP11B1* gene from the four unrelated Chinese patients are identical (**Figure 3**) (29, 32), suggesting that it is a founder rearrangement event in Chinese population. To confirm its founder effect, we used our optimized CNV detection algorithm to recall in-house 10000 WES (whole-exome sequencing) data. Two additional heterozygous carriers with the *CYP11B2/CYP11B1* chimeric gene with identical breakpoints were validated by our breakpoint-specific PCR and Sanger sequencing (**Figures 1G**, **3**), suggesting that the allele frequency of this *CYP11B2/CYP11B1* chimeric gene is approximately 1/10000 in Chinese population. Private communication with one commercial genetic testing company also suggested this prevalence because six carriers were detected in their 30000 WES dataset, although without validation experiments.

Patients with 11β-OHD can be diagnosed in early childhood due to ambiguous genitalia and accelerated growth in females; however, precocious puberty or advanced bone age was not easily observed in males, so the diagnosis was generally made later in males. In our study, patient 1 and patient 3 were diagnosed at the age of 4-6 years with Tanner stage II and significantly advanced bone age, and both of their final heights were already severely impaired at that time. Through drug treatment immediately after diagnosis, the conditions of all three patients improved. Their growth velocity declined with deceleration of their bone maturation, and adrenal androgen levels remained stable. Moreover, secondary sexual development regressed in patients 1 and 3, and no further clitoral enlargement was observed in patient 2. Blood pressure was well controlled by hydrocortisone replacement for patient 2 and by a combination of blood pressure medications such as spironolactone for patients 1 and 3. Therefore, early diagnosis and reasonable treatment are important to prevent complications and improve outcomes.

In conclusion, our optimized target sequencing can detect the point mutation, intragenic/intergenic deletion and even the chimeric *CYP11B2/CYP11B1* gene, and improved the genetic diagnosis of 11β-OHD in a one-tube test. In addition to one novel indel, we confirmed the chimeric *CYP11B2/CYP11B1* gene in intron 6 of *CYP11B2* as a founder rearrangement event in Chinese population.

## Data Availability Statement

All data have been deposited in the Chinese National Genomics Data Center (https://ngdc.cncb.ac.cn/omix) with accession number HRA002372.

## Ethics Statement

The studies involving human participants were reviewed and approved by the ethics committee of the Capital Institute of Pediatrics (SHERLL 2018005). Written informed consent to participate in this study was provided by the participants’ legal guardian/next of kin. Written informed consent was obtained from the individual(s), and minor(s)’ legal guardian/next of kin, for the publication of any potentially identifiable images or data included in this article.

## Author Contributions

HX, XLC, and XBC designed the study. HX, HY, and XLC wrote and revised manuscript. HX and XLC were responsible for target sequencing data analyses. HY, XY, and XBC were responsible for clinical works including diagnosis and revisit of patients. YZ finished the target sequencing and long-range PCR. NL was responsible for analysis of CNVs and breakpoint using target sequencing data. All authors contributed to the article and approved the submitted version.

## Funding

This work was supported by grants from the Beijing Natural Science Foundation (7202019 to XLC), the Beijing Municipal Administration of Hospitals Incubating Program (PX2020056 to HX), Capital Health Research and Development of Special (2018-2-2101 to XBC, 2020-1-4071 and 2020-2-1131 to XLC), the Capital Institute of Pediatrics foundation (PY-2019-05 to Shaofang Shangguan) and Research Foundation of Capital Institute of Pediatrics (CXYJ-2021006 to XLC).

## Conflict of Interest

Author NL was employed by Beijing Mygenostics Co.,Ltd.

The remaining authors declare that the research was conducted in the absence of any commercial or financial relationships that could be construed as a potential conflict of interest.

## Publisher’s Note

All claims expressed in this article are solely those of the authors and do not necessarily represent those of their affiliated organizations, or those of the publisher, the editors and the reviewers. Any product that may be evaluated in this article, or claim that may be made by its manufacturer, is not guaranteed or endorsed by the publisher.
