# Supplementary material for: Detection of Small CYP11B1 Deletions and One Founder Chimeric CYP11B2/CYP11B1 Gene in 11β-Hydroxylase Deficiency
Source: Front Endocrinol (Lausanne). 2022 May 24;13:882863. doi: 10.3389/fendo.2022.882863 (PMC9171383; doi:10.3389/fendo.2022.882863)
Supplement: Supplementary file 1 [file DataSheet_1.docx]

Supplementary Table 1. Gene list in target sequencing panel

| *A2ML1* | *AAAS* | *AARS2* | *ABCD1* | *AIP* | *AIRE* | *AKR1C2* | *AKR1C4* |
| --- | --- | --- | --- | --- | --- | --- | --- |
| *AMH* | *AMHR2* | *ANOS1* | *APC* | *AQP2* | *AR* | *ARL6* | *ARMC5* |
| *ARNT2* | *ARX* | *ATRX* | *AURKC* | *AVP* | *AVPR2* | *BBS1* | *BBS10* |
| *BBS12* | *BBS2* | *BBS4* | *BBS5* | *BBS7* | *BBS9* | *BMP15* | *BMP4* |
| *BMPR1B* | *BRAF* | *BSND* | *BTK* | *CASR* | *CATSPER1* | *CBX2* | *CCDC28B* |
| *CD96* | *CDKN1B* | *CDKN1C* | *CDON* | *CEP19* | *CEP290* | *CFTR* | *CHD7* |
| *CHEK2* | *CHRM3* | *CLCNKA* | *CLCNKB* | *CLPP* | *CYB5A* | *CYP11A1* | *CYP11B1* |
| *CYP11B2* | *CYP17A1* | *CYP19A1* | *CYP21A2* | *DAZL* | *DCAF17* | *DHCR7* | *DHH* |
| *DIAPH2* | *DISP1* | *DMRT1* | *DPY19L2* | *DUSP6* | *ERCC6* | *ERCC8* | *ESR1* |
| *FEZF1* | *FGD1* | *FGF17* | *FGF8* | *FGFR1* | *FGFR2* | *FIGLA* | *FLRT3* |
| *FMR1* | *FOXL2* | *FSHB* | *FSHR* | *GATA4* | *GDNF* | *GH1* | *GH2* |
| *GHR* | *GHRH* | *GHRHR* | *GHSR* | *GK* | *GK2* | *GLCCI1* | *GLI2* |
| *GLI3* | *GNAI2* | *GNAS* | *GNRH1* | *GNRHR* | *GOPC* | *GPR101* | *H19* |
| *H6PD* | *HARS2* | *HCCS* | *HDAC8* | *HESX1* | *HFE* | *HFM1* | *HGF* |
| *HOXA13* | *HS6ST1* | *HSD11B1* | *HSD11B2* | *HSD17B3* | *HSD17B4* | *HSD3B2* | *IARS2* |
| *ICK* | *IGSF1* | *IL17RD* | *INSL3* | *INSR* | *IRF6* | *KCNJ1* | *KCNJ5* |
| *KCNQ1OT1* | *KDM6A* | *KIF1B* | *KISS1* | *KISS1R* | *KLHL10* | *KMT2D* | *KRAS* |
| *LARS2* | *LEPR* | *LHB* | *LHCGR* | *LHX3* | *LHX4* | *LZTFL1* | *MAMLD1* |
| *MAP2K1* | *MAP2K2* | *MAP3K1* | *MAX* | *MC2R* | *MCM4* | *MCM9* | *MED12* |
| *MEN1* | *MID1* | *MKKS* | *MKRN3* | *MKS1* | *MRAP* | *MYH8* | *NAA10* |
| *NANOS1* | *NF1* | *NFKB2* | *NNT* | *NOBOX* | *NR0B1* | *NR3C1* | *NR5A1* |
| *NRAS* | *NSDHL* | *NSMF* | *ORC1* | *OTX2* | *PAX6* | *PCNT* | *PCSK1* |
| *PDE11A* | *PDE8B* | *PEX1* | *PEX10* | *PEX12* | *PEX13* | *PEX14* | *PEX19* |
| *PEX2* | *PEX26* | *PEX3* | *PEX5* | *PEX6* | *PHF6* | *PLAU* | *POF1B* |
| *POLR3A* | *POLR3B* | *POMC* | *POR* | *POU1F1* | *PRKACA* | *PRKAR1A* | *PRKCA* |
| *PROK2* | *PROKR2* | *PROP1* | *PSMC3IP* | *PTCH1* | *PTPN11* | *RAB23* | *RAB3GAP2* |
| *RAF1* | *RASA2* | *RBM28* | *REN* | *RET* | *RIPK4* | *RIT1* | *RNF216* |
| *ROR2* | *RSPO1* | *RXFP2* | *RXRA* | *RXRB* | *SDCCAG8* | *SDHB* | *SDHC* |
| *SDHD* | *SEMA3A* | *SEMA3E* | *SHH* | *SHOC2* | *SIX3* | *SLC12A1* | *SLC26A8* |
| *SOS1* | *SOX10* | *SOX2* | *SOX3* | *SOX9* | *SPATA16* | *SPRY4* | *SRD5A2* |
| *SRY* | *STAG3* | *STAR* | *STAT5B* | *SYCP3* | *TAC3* | *TACR3* | *TAF4B* |
| *TBX19* | *TGIF1* | *THRA* | *THRB* | *TMEM127* | *TMEM67* | *TP53* | *TRH* |
| *TRHR* | *TRIM32* | *TSPYL1* | *TTC8* | *TWNK* | *TXNRD2* | *USP9Y* | *UTY* |
| *VHL* | *WDPCP* | *WDR11* | *WNK1* | *WNK4* | *WNT3* | *WNT4* | *WNT5A* |
| *WT1* | *ZFPM2* | *ZIC2* | *ZMYND15* |  |  |  |  |

Supplementary Table 2. Primers Used for PCR Assay of *CYP11B1* speculated arrangements

| Patient ID | Primer | Sense strand | Antisense strand | Fragment size (bp) |
| --- | --- | --- | --- | --- |
| P1 | *CYP11B2/CYP11B1* | TGTCCCCACTGGAAAGCTCT | ACGCTCCTCACCATACCAAC | 1639 |
| P2 | *CYP11B1*  Exon 1-3 del | TGTCTGGTATTTCCCGTGCT | GTGTTTATCACATCACAATCCCAAGT | 1497 |
| P3 | *CYP11B1*  Exon 3-4 del | CAGAAAATCCCTCCCCCCTA | GTGTTTATCACATCACAATCCCAAGT | 1467 |

Supplementary Table 3. Primers Used for PCR Assay of *CYP11B1* Gene

| Primer | Sense strand | Antisense strand | Fragment size (bp) |
| --- | --- | --- | --- |
| *CYP11B1*-exon 1-2 | CTCTCGAAGGCAAGGCACCAG | CTGCTCCCAGCTCTCAGCTCG | 878 |
| *CYP11B1*-exon 3-5 | CAGAAAATCCCTCCCCCCTA | GTGTTTATCACATCACAATCCCAAGT | 1467 |
| *CYP11B1*-exon 6-9 | TGACCCTGCAGCTGTGTCTC | CCCTGGGTGCAGAGACGT | 1552 |
