# Supplementary material for: Detection of Small CYP11B1 Deletions and One Founder Chimeric CYP11B2/CYP11B1 Gene in 11β-Hydroxylase Deficiency
Source: Front Endocrinol (Lausanne). 2022 May 24;13:882863. doi: 10.3389/fendo.2022.882863 (PMC9171383; doi:10.3389/fendo.2022.882863)
Supplement: Supplementary file 2 [file DataSheet_2.docx]

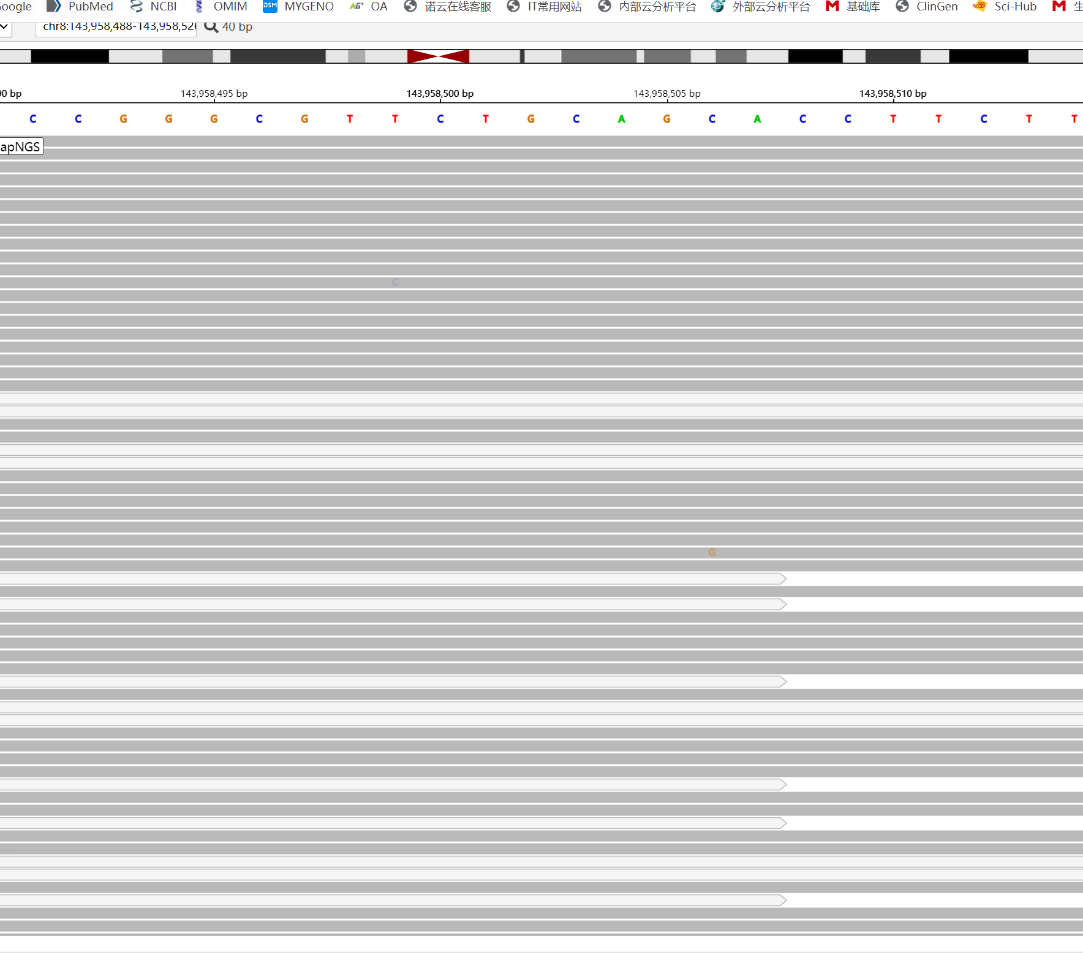


**Supplementary Figure 1** Misalignment information of split reads indicative breakpoints of *CYP11B1* deletion in patient 2 identifying by IGV. The orange arrow indicated the breakpoint of chr8:143958508 (hg19).

**A**


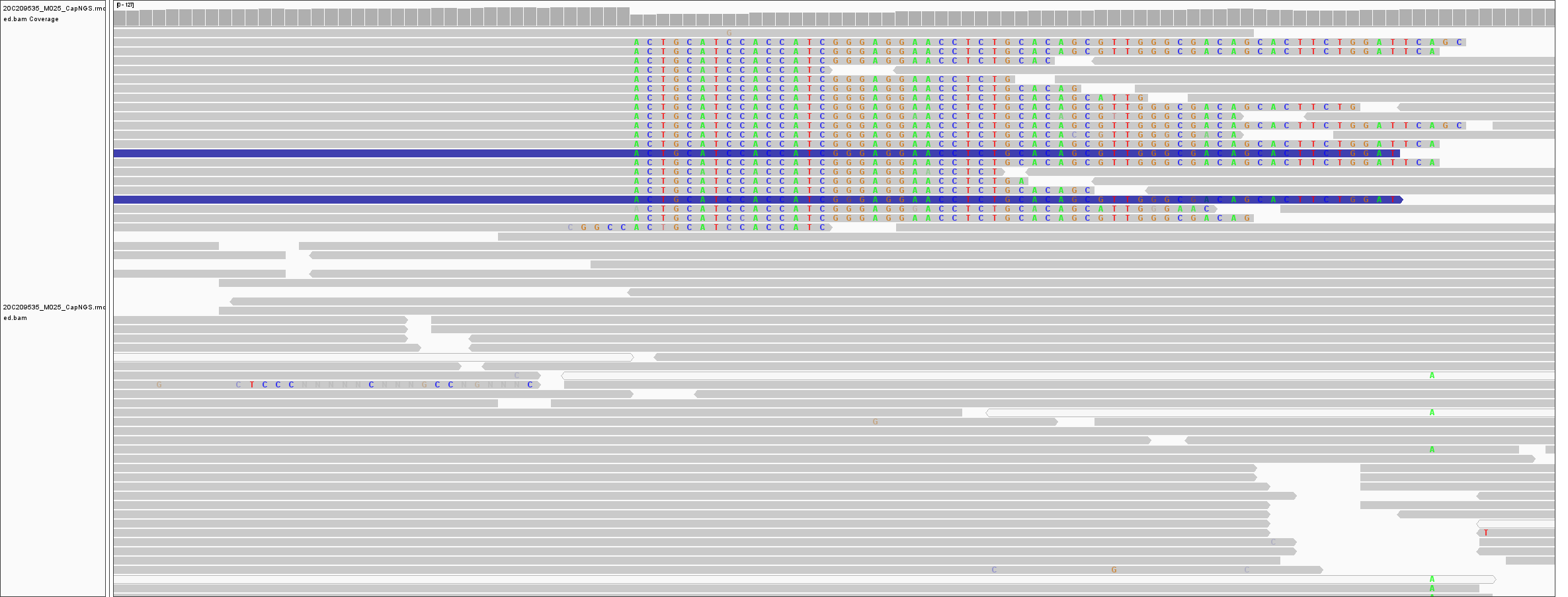


**B**


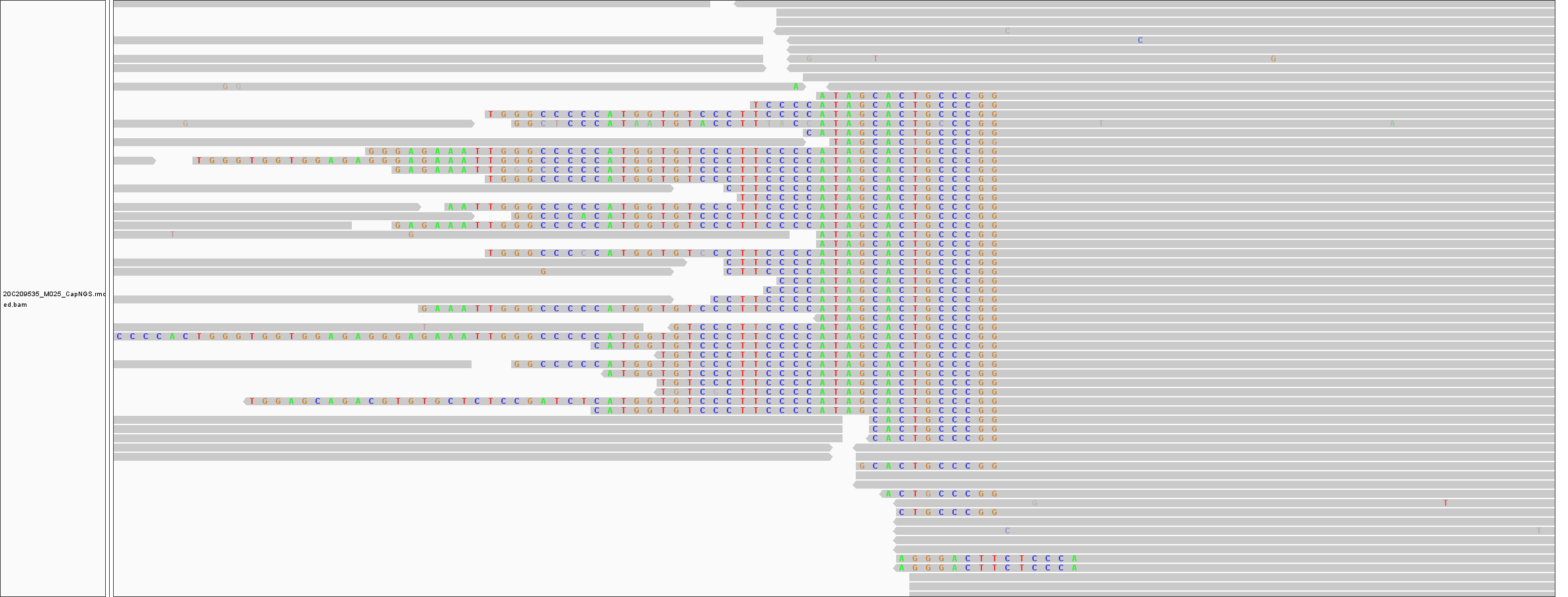


**Supplementary Figure 2** Misalignment information of split reads indicative breakpoints of *CYP11B1* deletion in patient 3 identifying by IGV. The orange arrow indicated the breakpoint of chr8:143958084 (**A**) and chr8:143958532 (**B**).
